# Editorial: Phage Therapy: Past, Present and Future

**DOI:** 10.3389/fmicb.2017.00981

**Published:** 2017-06-15

**Authors:** Stephen T. Abedon, Pilar García, Peter Mullany, Rustam Aminov

**Affiliations:** ^1^Department of Microbiology, The Ohio State UniversityMansfield, OH, United States; ^2^Spanish National Research CouncilVillaviciosa, Spain; ^3^Department of Microbial Diseases, Eastman Dental Institute, University College LondonLondon, United Kingdom; ^4^School of Medicine and Dentistry, University of AberdeenAberdeen, United Kingdom

**Keywords:** bacteriophage therapy, bacterial infection treatment, biofilms, immunology, lysins, biocontrol, regulation

## Introduction

As an ancient proverb states, “The enemy of my enemy is my friend.” The so-called strictly lytic or virulent bacteriophages (phages)—especially the viruses of pathogenic bacteria—can certainly be considered enemies of “bad” bacteria and thereby our friends. The phage potential as antibacterial agents was recognized almost immediately upon the first generally accepted descriptions of these viruses as transmissible bacteriolytic entities (Abedon et al., 2011). As this was prior to Fleming's (1929) discovery of naturally occurring antibiotics, rather than being named as variations on that theme, the Greek concept of “phage” was chosen instead (d'Hérelle, 1917). “Phage” seemingly is a description of the macroscopic impact these viruses have on bacteria, which to the eye appear to be “eaters” or “devourers” of bacterial *cultures* (Summers, 1991), in broth or solid media.

The therapeutic, antibacterial application of phages came to be known as phage therapy, especially in clinical or veterinary contexts. More broadly, phages have also been used as biological control agents, reducing bacterial loads in foods, e.g., such as of *Listeria monocytogenes* in food processing (Bai et al., 2016), of zoonotic pathogens in food animals (Atterbury, 2009), or, in the treatment of crops against plant pathogenic bacteria as reviewed by Buttimer et al. Furthermore, modified phages can be used as DNA, protein, or drug delivery vehicles (Clark et al., 2012), and non-bacterial viruses can be used as biological control agents as well (e.g., Hyman et al., 2013; Kondo et al., 2013; Gilbert et al., 2015). Phage study, whether ultimately for therapy or biocontrol, spans from purely clinical observation to molecular analysis to considerations of immunology as well as ecology, the latter as phages represent essentially “living” drugs. In addition is the development of enzybiotics, which are therapeutic enzymes and most prominently include phage endolysins. The latter are proteins which phages employ to lyse the bacteria they are infecting, thereby releasing intracellularly produced phage progeny (Fischetti, 2008).

This diversity of studies and approaches to antibacterial therapy is important since, despite ~100 years of phage and phage therapy study (Abedon et al., 2011), there is still much to learn about phages and their use as therapeutic agents. There is also a compelling need for new safe and effective selectively toxic antibacterials, especially in the face of the antibiotic resistance crisis (Aminov, 2010). Phages and their products thus represent a largely untapped supply of such antimicrobials. Their use, however, has not yet been broadly embraced by the modern medical establishment. Exceptions are found especially in the countries of Georgia, Poland, and Russia, where phage therapy has been practiced by clinicians for many decades (Kutter et al., 2015; Cooper et al.).

**Figure 1:**
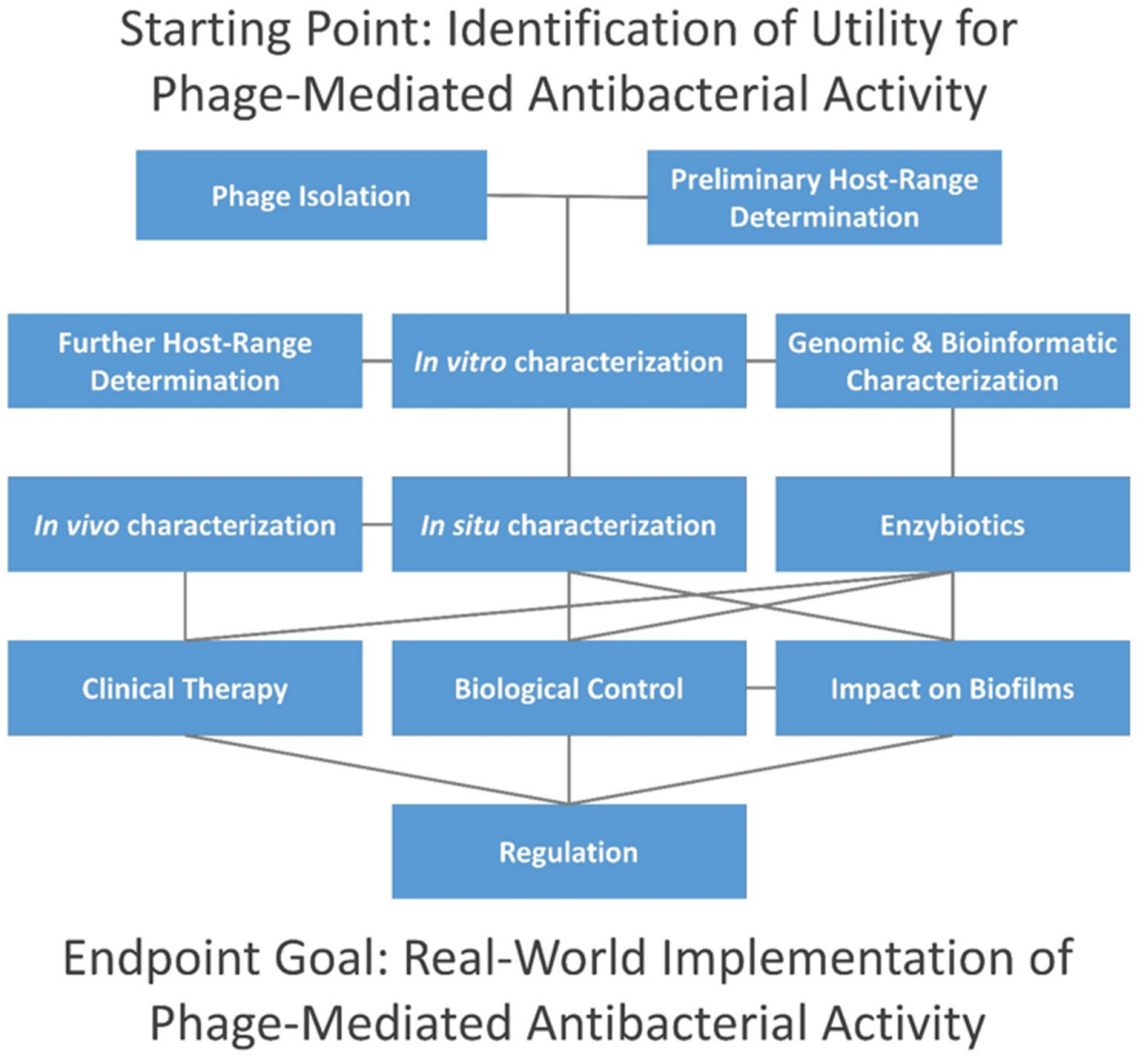
Topics addressed in this editorial. Connections are indicated via horizontal, vertical, and diagonal lines, and initial steps are found at the top of the figure. Consideration of time and resources required by each step is beyond the scope of this editorial, though individual aspects are considered in articles as cited in the main text. In summary, phage isolation is typically done in combination with preliminary host-range characterization, i.e., as in terms of enrichment and isolation hosts. This is followed by *in vitro* characterization in association with further host-range characterization (i.e., involving a larger panel of potential hosts) and bioinformatic (*in silico*) characterization. Enzybiotic development, if undertaken, typically will follow host-range and *in silico* characterization. For promising phages, *in situ* characterization comes next, including animal models for potential human treatments (*in vivo* characterization), or with other species for non-human treatments. Clinical testing can follow, including treatment of non-human species. Alternatively, phages may be employed for biological control of environments, and both biological control and therapeutic use of phages can be against biofilms. Not only may whole phages be used for therapy or control but so too may enzybiotics. Further development toward successful commercial or public-sector implementation generally must address regulatory requirements.

In this topic, we present 37 articles on or related to the use of lytic phages as antibacterial agents. These are grouped into several distinct categories, including (i) phage isolation for phage therapy, (ii) host range characterization, (iii) other *in vitro* phage characterizations, (iv) *in vivo* phage characterization, (v) characterization of phage *therapy* in animals, (vi) phage impact on bacterial biofilms, (vii) enzybiotics, (viii) clinical phage therapy, (ix) biological control of bacteria using phages, and (x) the current state of phage therapy implementation (Figure 1).

## Phage isolation for phage therapy

Key to any successful drug development is its discovery and subsequent characterization. For phage therapy, equivalent steps should be taken, including determination of how to combine phages into multi-phage mixtures known as phage cocktails. The review article in this topic by Weber-Dąbrowska et al. discusses the essential steps involved including sources and methods of phage isolation, choice of phage-propagation hosts, methods of characterization, selection criteria for therapeutic purposes, and limitations on phage procurement for therapy.

The use of phages as antibacterial therapeutics is especially important for targeting those pathogens for which antibiotic treatment options are limited. On-demand isolation of corresponding phages can be achieved via the enrichment of samples from environmental reservoirs, as explored by Mattila et al. Interestingly, the efficiency of enrichment-based phage isolation from municipal sewage varies considerably, with the best results seen for *Pseudomonas aeruginosa, Salmonella*, and the extended spectrum β-lactamase (ESBL) producing *Escherichia coli* and *Klebsiella pneumoniae*. The procedure is less efficient for vancomycin-resistant *Enterococcus* and *Acinetobacter baumannii*, while isolation of new phages against methicillin-resistant *Staphylococcus aureus* (MRSA) strains was very difficult. Potentially, the latter may be due to the choice of environmental reservoir used for the anti-MRSA phage isolation since, as Wang et al. show, pig fecal sewage may be a better source for these phages.

## Host range characterization

Prior to animal testing there are various approaches toward characterizing phages for antibacterial effectiveness (Weber-Dąbrowska et al.). Most important is the range of bacteria targeted (Mirzaei and Nilsson, 2015). As a minimal requirement for phage therapy, a phage should be able to infect the bacterial isolates it is supposed to be targeting, and to display reasonable specificity so that non-target bacteria are not affected. A proper understanding of phage host range is also necessary for the development of efficient cocktails, which ideally would be formed using multiple phages that possess synergistic properties, particularly in terms of host range, thereby offering better infection control capability. Nevertheless, for some phage applications such as phage therapy and phage-based biosensors, it should be taken into account that host range is not a fixed property, but rather it can evolve over time, thereby changing phage specificity (Ross et al., 2016).

For obvious reasons, multidrug-resistant (MDR) pathogens are a primary target for phage therapy. The host range of four phage cocktails that are approved and commercially available in Georgia have been tested by Gundogdu et al. on a panel of 142 clinical strains of *E. coli* isolated in Turkey and possessing extended-spectrum β-lactamase activity. The phage cocktail antibacterial efficiencies varied from 59.2 to 87.3% of strains, as based on spot testing, which is promising given that these were difficult-to-treat MDR bacterial strains.

In addition, and like antibiotic therapy, phage therapy can result in the evolution of bacterial resistance. Understanding resistance development is important in terms of both basic biology and phage-based applications. Some phage resistant bacteria are less fit than their phage-sensitive parents. Lim et al. found that phage PB1-resistant *P. aeruginosa* displayed small-colony variants which were impaired in biofilm formation, were more antibiotic sensitive, displayed decreased twitching motility, and had reduced elastase and pyocyanin production.

## Other *In vitro* phage characterizations

In addition to the assessment of host range (previous section), other phage “organismal” characteristics such as burst size, ability to display lysogeny, and general plaque morphology should be evaluated. *In vitro* characteristics also include the ability to degrade experimental bacterial biofilms (subsequent section) along with complete genome sequencing. The latter typically is followed by *in silico* analyses, especially to exclude phages carrying bacterial virulence factor genes. Also, it is advantageous to exclude phages carrying lysogeny-associated genes. Hamdi et al. isolated five phages that infect *Citrobacter freundii* which they found had no known virulence factor or integrase genes. The latter are employed by many phages to initiate lysogenic cycles. Such properties suggest potential utility for these phages as antimicrobial agents.

Bardina et al. isolated and characterized three phages (UAB_Phi20, UAB_Phi78, and UAB_Phi87) infecting *Salmonella* to reduce the presence of this zoonotic bacterium in poultry. Sequence analysis of genomes did not indicate the presence of virulence factor or antibiotic resistance genes. Phage UAB_Phi20, however, encodes lysogeny-associated genes, although no lysogens could be isolated. The authors suggest that this could be because of a lack of signals needed to transcribe the CI repressor gene required for establishment of lysogeny.

Lytic phage development also depends on the physiological state of the host. According to Bryan et al. T4 phages infecting stationary phase *E. coli* may enter a “hibernation” mode, which is a persistent but reversible dormant state. Infected bacteria continue to produce some phage proteins, but phage development is halted until appropriate nutrients become available. A “scavenger” mode is encountered when exposed to limited nutrients, with the production of small quantities of progeny per infection. These considerations are important in understanding phage therapy of bacteria displaying varied physiological states, such as within biofilms or during chronic bacterial infections.

## *In vivo* phage characterization

By *in vivo* we mean *in situ* phage assessment within other organisms or surrogates, such as during animal testing (further considered in a subsequent section). Such assessment includes in terms of safety to the host during treatment, though in practice few side effects with phage therapy have been detected (Miedzybrodzki et al.). Potential cytotoxic effects can also be evaluated using eukaryotic cell lines via different assays such as trypan blue, staining with Hoechst and propidium iodide, lactate dehydrogenase release, and the MTS (3-(4,5-dimethylthiazol-2-yl)-5-(3-carboxymethoxyphenyl)-2-(4-sulfophenyl)-2H-tetrazolium) assay, as described in this topic by Henein et al.

Important as well are phage and especially virion interactions with immune systems, which is a concern for biologics generally, i.e., protein-based drugs. In the article by Mirzaei et al. several *E. coli* phage preparations, were found to induce strong cytokine-driven inflammatory responses in HT-29 and Caco-2 intestinal epithelial cells. Whether this was the effect of phages *per se* or residual contaminants in phage preparation(s) was questioned by Dufour et al., however. As a Response, Mirzaei et al. proposed morphological differences as possible bases of contradictory outcomes, perhaps highlighting a need for better standardization of approaches. Mirzaei et al., in a subsequent Corrigendum, acknowledged that at least some aspect of the cytokine responses described in the original publication may have been due to residual contaminants.

For lipopolysaccharide (LPS)-activated monocytes, neither purified phage T4 nor T4 lysate, according to the results of Bocian et al., had a significant impact on the *ex vivo* human immune response. Phage lysates however, may affect the differentiation of human monocytes into myeloid dendritic cells, but purified phage preparations do not have that effect (Bocian et al.). Also regarding LPS, Miernikiewicz et al. found that recombinant short tail fiber (gp12) from phage T4 decreased inflammatory responses to LPS in a murine model. Cell culture and mouse testing indicated no toxicity, suggesting that this recombinant protein potentially could be used as an anti-LPS medicinal.

No significant increase in antiphage antibodies in the sera of most patients undergoing anti-staphylococcal phage therapy were detected by Żaczek et al. In patients with the increased titers of antiphage IgG and IgM to these phages, no interference with phage therapy clinical outcomes were observed. While the influence of purified T4 and A3/R phages on differentiation of human myeloid dendritic cells (DCs) from monocytes is negligible, phage-lysed bacterial material has a substantial effect on their differentiation (Bocian et al.). Thus, the products of phage-induced lysis of bacteria during phage therapy could influence the differentiation as well as potentially the functions of DCs that are differentiating from monocytes recruited to sites of infection.

## Characterization of phage therapy in animals

In the modern era, clinical use of drugs typically is preceded by animal testing. Phage therapy, since it has been in practice for so long, comes from a tradition where clinical use has tended to take precedence over animal testing (Abedon, 2015c). Phage therapy in the modern era nonetheless has to adopt current standards of drug development, that is, in which animal testing by necessity precedes clinical use, and several articles in the topic are devoted to animal testing of phages and phage preparations.

Wang et al. characterized the staphylococcal phage SLPW. Treatment of intra-abdominal MRSA infections in mice with phage SLPW provided high protection (80% survival) as well as reduction of infection-induced inflammatory cytokines, thus substantiating this phage as a potential therapeutic agent against MRSA infections. With a *Clostridium difficile* target, a 4-phage cocktail was tested in a *Galleria mellonella* larva model and was found by Nale et al. to be as effective as vancomycin. Another problematic multi-drug resistant nosocomial pathogen, *A. baumannii*, was targeted using phage vB-GEC_Ab-M-G7 by Kusradze et al. In a rat wound model, this phage substantially decreased bacterial loads.

Abedon briefly reviewed in a general commentary a rabbit staphylococcus osteomyelitis model system published by Kishor et al. (2016). Presented as well is a summary of several animal presumptive chronic infection models previously used for phage therapy development. A series of criteria are suggested for confirmation that such systems represent adequate disease models including demonstration of antibiotic tolerance by infecting bacteria and/or of presence of biofilms.

Pharmacological issues of phage therapy include phage transit from the stomach to the distal gastrointestinal tract. Międzybrodzki et al. showed in a rat model that modification of the stomach environment using the drugs ranitidine and omeprazole, which reduce production of stomach acid, protect staphylococcal phage A5/80, allowing passage to the lower intestine. These authors also found that phage penetration from oral administration to systemic circulation can differ among phage types as phage A5/80 reaches the bloodstream following oral administration aided by acid-reducing drugs but similarly administered T4 did not.

## Phage impact on bacterial biofilms

Formation of biofilms during bacterial infection is one of the major problems in infection control. Bacteria in biofilms are extremely resistant to antimicrobials, well protected from host defenses, and tend to develop chronic infections (Cooper et al., 2014). Some bacteriophages penetrate biofilms and this may supplement or replace a less efficient antibiotic treatment (Abedon, 2015a,b). *C. difficile*, for example, produces biofilms which contribute to its virulence and impair antimicrobial activity. Nale et al. found that a cocktail of *C. difficile* phages could significantly reduce these biofilms and prevent colonization when used either alone or in combination with vancomycin.

Catheter-associated urinary tract infections (CAUTIs) such as caused by *Proteus mirabilis* are very difficult to treat as they form biofilms that are highly tolerant to antibacterials. Two novel virulent phages active against *P. mirabilis* were isolated, characterized, and studied for application on catheter-associated biofilms by Melo et al. In a dynamic biofilm model simulating CAUTIs, the authors demonstrated a significantly lower rate of *P. mirabilis* biofilm formation up to 168 h following catheterization, thus highlighting the potential of these phages in preventing bacterial surface colonization.

Biofilms can also be targeted by degrading the matrix in which bacterial cells are suspended. Gutiérrez et al. tested a recombinant protein from a staphylococcal phage encoding an exopolysaccharide depolymerase, a kind of enzybiotic. In polysaccharide producing staphylococci the enzyme can prevent and disperse biofilms, thus potentially allowing better antimicrobial access to targeted bacteria.

## Enzybiotics

Purified antibacterial enzymes have been described as enzybiotics (Veiga-Crespo et al., 2007), i.e., as derived from ‘antibiotic’. These can include extracellular polymeric substance (EPS) depolymerases (as above) but also, phage-encoded lytic enzymes, i.e., lysins. Though some lysins are virion-particle associated, as are many EPS depolymerases (Pires et al., 2016), the majority are endolysins, meaning “from-within cell-wall degrading enzymes.” Enzybiotics upon purification, however, are applied from without.

The peptidoglycan of Gram-positive bacteria is not protected by an outer membrane so is directly susceptible to phage lysins applied from without. Blazquez et al. generated a novel (“tailor-made”) endolysin (PL3) targeting *Streptococcus pneumoniae*. It combines the amidase activity of a phage endolysin (Pal) with that of LytA, a *Streptococcus* autolysin. Joining these two unrelated catalytic domains into a single protein resulted in greater antibacterial activity in a zebrafish model.

In Gram-negative bacteria, phage lysins typically need to be modified to penetrate the outer membrane barrier. This can be done by engineering hybrid molecules that combine natural lysin with an antimicrobial peptide. Yang et al. found that one such construct, PlyA, displayed good activity against growing cultures of both *A. baumannii* and *P. aeruginosa*, but not against stationary phase cells unless used with outer membrane permeabilizing agents. No antibacterial activity, however, could be detected in some bio-matrices such as culture media, milk, or sera, suggesting a need for further optimization. Endolysins such as ABgp46, as characterized by Oliveira et al., are also active against *A. baumannii*, including MDR strains. In addition, the range of activity of this lysin can be extended to other Gram-negative bacteria if used in combination with outer membrane permeabilizing agents. Endolysin LysABP-01 from *A. baumannii* phage ØABP-01 also possesses antibacterial activity against *A. baumannii* and *P. aeruginosa* as well as *E. coli*, which as shown by Thummeepak et al. can be enhanced in the presence of the antibiotic colistin.

## Clinical phage therapy

Clinical phage therapy is the treatment or prevention of infections in humans and the use of phages in microbiome modification. In addition is the related use of phages to treat or prevent infections in animals. Clinical phage therapy is permitted for routine use in a limited number of countries though the corresponding data from these efforts is limited. Because of the long-term treatment requirements of chronic conditions such as cystic fibrosis, the appearance of bacterial resistance to phages can be a problem. Krylov et al. propose to employ a combinatorial approach during treatment of drug-resistant *P. aeruginosa* to circumvent this problem, by using phages with a proven safety record combined into cocktails.

Although not life-threatening, some chronic skin infections, such as caused by *Propionibacterium acnes*, can require long-term antibiotic treatment, thus contributing to dysbiotic changes in microbiomes and selection for antimicrobial resistance. Phage therapy of acne may be a valuable alternative to reduce the overuse of antibiotics in the treatment of this condition, as reviewed by Jonczyk-Matysiak et al.

## Biological control of bacteria using phages

In the review article by Buttimer et al., phage biocontrol of bacterial crop diseases is compared to chemical control measures. Phages they suggest are more environmentally friendly, can be tailored against specific disease-causing bacteria, and can be easily reformulated if resistance develops. Some field trials, for example, have shown potential for phage biocontrol of bacterial blight of leek, as explored by Rombouts et al.

Another aspect of biocontrol (vs. phage therapy in the strictest sense) is reduction of loads in animals of what otherwise could be food-borne pathogens. In poultry, Ahmadi et al. found that the prophylactic administration of phage PSE, active against *Salmonella enterica* serovar Enteritidis, significantly reduced shedding of this pathogen. Improved biocontrol measures against *Salmonella* will include the selection of phages that can infect a broader range of bacterial strains. This has been explored, including in terms of phage genomics, by Bardina et al.

In another example of biocontrol, Hernández reported that bacteriophages against *Serratia* spp., which can spoil Atlantic horse mackerel (*Trachurus trachurus*), were isolated and tested for protection of fresh filets. Reductions in *Serratia* counts of more than 90% were observed in treatment with about 10^8^ phages per gram of filet after 6 days of refrigerated storage (6°C). Phage application at lower densities was less effective.

## The current state of phage therapy implementation

An important aspect of drug utility is availability, and this requires adequate manufacturing, marketing, and delivery to as well as education of users. Prior to these steps, it is necessary to maintain an adequately robust development pipeline along with strategies toward regulatory approval. Presently, phage therapy is relatively extensively used only in three countries, Georgia, Poland, and Russia, while its acceptance and re-implementation in other countries is still pending (Expert Round Table on Acceptance and Re-Implementation of Bacteriophage Therapy, 2016). As discussed by Cooper et al., difficulties with acceptance are due to: (i) differences in biological, physical, and pharmacological properties of phages compared to conventional antimicrobials, (ii) the need to employ multiple phage isolates (cocktails) due to the high specificity of phages (thereby allowing for more effective presumptive treatment, that is, treatment which is initiated prior to precise diagnosis of microbial etiologies), and (iii) current approval processes for antimicrobial agents that are based on chemically derived drugs and which consequently are less suitable for phages. Alternative approval pathways may be required for phage therapy (Aminov et al., 2017), while phage-derived enzybiotics are already suitable for the current approval processes as therapeutic proteins.

Based on the wealth of data obtained by some phage therapy centers, Górski et al. suggest that it is time to consider phage therapy benefits in their entirety, including compassionate use targeting cohorts of patients for whom no alternative treatment is currently available. The effects of phage therapy, which target infectious agents and which also can modulate the immune system, resemble the effect of antibiotics, which, in addition to antibacterial activity, can display other regulatory effects on the human body (Aminov, 2013). Thus, the impact of phages beyond intended antibacterial activity should be carefully evaluated in association with more standard practices of phage therapy development. Lastly, Nagel et al. point out that as infectious diseases significantly affect developing countries, phage therapy considering its relative technical simplicity as well as ease of phage isolation, characterization, and production, could be especially useful in these settings.

## Conclusion

Bacteriophages have been instrumental in the development of modern biology, particularly the understanding of biological process at the molecular level which has been crucial for the development of modern biological sciences (Cairns et al., 1966; Summers, 1999). They have also been used therapeutically for ~100 years, with a good safety record (although their exploitation in this regard has lagged behind their use in molecular biology). Publications demonstrating the safety of phage applications (some of which include phase I safety trials) include (Rhoads et al., 2009; Wright et al., 2009; Miedzybrodzki et al., 2012; Sarker et al., 2012, 2016; Rose et al., 2014; Fish et al., 2016; Speck and Smithyman, 2016). Nonetheless, and despite a demonstrated need for new, safe antibacterial agents (Aminov, 2016), phage use by most Western physicians has not yet caught on, and this is due (presumably) to a lack of familiarity with phage therapy, but also because of a relative lack of regulatory approval. This volume provides an overview of a substantial number of facets of use of phages and their products in a medical and especially antibacterial context. We believe, in the face of the looming antibiotic crisis, that this approach deserves serious consideration. Hopefully phages can prove as revolutionary in the medical field as they have in the scientific.

## Author contributions

All authors listed have made substantial, direct and intellectual contribution to the work, and approved it for publication.

### Conflict of interest statement

SA has industry ties but none were involved in this writing, and SA also is responsible for and promotes the websites phage.org and phage-therapy.org which deal with the topics similar to those presented here. The other authors declare that the research was conducted in the absence of any commercial or financial relationships that could be construed as a potential conflict of interest.
